# Cystic fibrosis related diabetes (CFRD) prognosis

**DOI:** 10.1016/j.jcte.2021.100278

**Published:** 2021-11-19

**Authors:** Zahrae Sandouk, Farah Khan, Swapnil Khare, Antoinette Moran

**Affiliations:** a Internal Medicine, Division of Metabolism, Endocrinology & Diabetes, University of Michigan, Ann Arbor, MI, USA; b Internal Medicine, Division of Metabolism, Endocrinology and Nutrition, University of Washington, Seattle, WA, USA; c Internal Medicine, Division of Metabolism, Endocrinology & Diabetes, Indiana University, Indianapolis, IN, USA; d Pediatric, Division of Pediatric Endocrinology, University of Minnesota, Minneapolis, MN, USA

**Keywords:** Cystic fibrosis, Cystic fibrosis related diabetes, Prognosis, Microvascular complications, Macrovascular complications, Transplant prognosis, CFTR modulators

## Abstract

Cystic fibrosis related diabetes (CFRD) occurs in at least 40–50% of adults with CF. With other forms of diabetes, microvascular and macrovascular disease are the major causes of morbidity and mortality. Macrovascular disease is rare in CF. While microvascular disease does occur in this population, there are CF-specific diabetes complications that have a more important impact on prognosis. The additional diagnosis of diabetes in CF is associated with decreased lung function, poor nutritional status, and an overall increase in mortality from lung disease. These negative findings start even before the clinical diagnosis of CFRD, during the period when patients experience abnormal glucose tolerance related to insulin insufficiency. The main mechanisms by which CFRD negatively affects prognosis are thought to be a combination of 1) protein catabolism, decreased lean body mass and undernutrition resulting from insulin insufficiency, and 2) an increased pro-inflammatory and pro-infectious state related to intermittent hyperglycemia. With the introduction of CFTR modulators, the care of CF patients has been revolutionized and many aspects of CF health such as BMI and lung function are improving. The impact of these drugs on the adverse prognosis related to the diagnosis of diabetes in CF, as well as the potential to delay or prevent onset of CFRD remain to be determined.

## Highlights

• Poor nutritional status and decreased lean body mass.
• Decline in pulmonary function.
• Increased mortality from lung disease.
• Microvascular complications.
• Macrovascular complications (not currently a significant complication but this may change with modulators).

## Prognosis of CFRD related to complications also common in types 1 and 2 diabetes (Fig. 1)

All forms of diabetes are associated with complications that affect prognosis. CFRD has some of these in common with type 1 (T1D) and type 2 (T2D), while other complications are unique to the CF population. The most common cause of death in T1D and T2D is cardiovascular disease (CVD). In contrast, CVD is rare in CFRD, and until recently was considered to be essentially non-existent even in patients in their 50′s and 60′s. The reasons for this “cardio-protective” effect of CF are unclear but have been postulated to be due to lean body habitus and unusually low cholesterol levels [1]. This picture may be changing, as discussed below, with new modulator therapies that help to reverse the basic CF chloride channel defect.

**Fig. 1:**
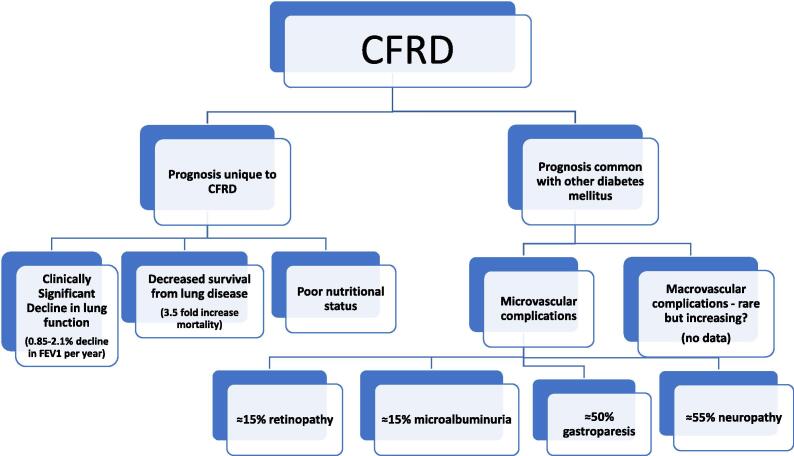
Overview of CFRD prognosis that is common to other diabetes mellitus and unique to CF.

Microvascular complications including retinopathy, neuropathy, nephropathy, and autonomic dysfunction are a significant source of morbidity in individuals with T1D, T2D as well as CFRD. However, they do not appear to progress to the same level of severity in CFRD. According to the CDC, in the US, diabetic nephropathy is the number one cause of kidney failure along with high blood pressure, and diabetic retinopathy is the leading cause of blindness in individuals aged 20–74 [2], [3]. In contrast, blindness and kidney failure (except for the lung transplant population, who receive nephrotoxic drugs) are exceptionally rare in people with CFRD, suggesting that the seriousness of microvascular complications is less in this population.

Despite less advanced microvascular disease in CFRD, the prevalence of *any* microvascular complications may be similar to that seen in T1D and T2D. Table 1 provides an overview. A comparative study that looked at data from a nationwide registry of diabetes in Germany and Austria noted that retinopathy and nephropathy did not occur at a statistically different rate in adults with CFRD compared to adults with T1D and T2D [4]. A study comparing 79 adults with CFRD to 79 adults with T1D (matched by age, sex, and duration of insulin therapy) found more microalbuminuria but less retinopathy in adults with CFRD. In this study, the prevalence of neuropathy and nephropathy were similar in both groups [5]. In another study, after 10 years or more of diabetes, 14% of CFRD patients had elevated urine microalbumin levels and 16% had mild retinopathy [6]. While total years duration of diabetes was counted in this analysis (years of early CFRD with normal fasting glucose levels plus years with fasting hyperglycemia), none of these patients had microvascular disease until diabetes had progressed to include fasting hyperglycemia. Autonomic neuropathy and gastrointestinal issues were seen in 52% of adults with CFRD, regardless of fasting hyperglycemia status [6], but the authors reported that some of these issues, such as delayed gastric emptying, are seen even in CF patients without diabetes and thus may be more related to the basic CF defect.

**Table 1:** Complications rates comparing CFRD, T1 and T2 DM. Source includes. Standards of Medical Care in Diabetes. 2021 report.

	CFRD	T1, T2DM
Microvascular complications Retinopathy Nephropathy Neuropathy Gastroparesis		
	15%	35%
	15%	20–40%
	55%	50%
	50%	5–12%
Macrovascular complications	No data	30%

While CF patients rarely progress to the level of blindness or kidney failure, microvascular complications do contribute to worsening health outcomes in adults with CFRD and validate the importance of regular and aggressive screening to minimize morbidity from these complications [7]. Annual screening for microvascular complications should start after a duration of five years with CFRD and fasting hyperglycemia [6]. While there are no reports on the impact of treatment on the development or progression of microvascular complications in adults with CFRD, given what we know about adults with T1D and T2D, early treatment intensification in adults with CFRD could very likely lead to long-term benefits and reduced progression of microvascular complications [8], [9].

## Prognosis of CFRD related to complications unique to diabetes in CF (Fig. 1)

### CFRD survival statistics

**Fig. 2:**
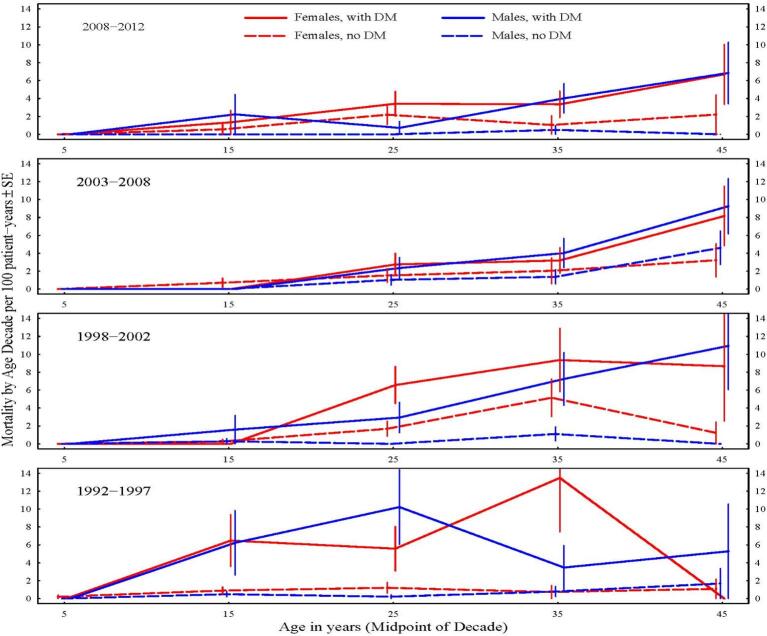
University of Minnesota mortality rates per 100 patient-years for cystic fibrosis (CF) patients with and without diabetes (DM) by sex and age decade over four time periods: 1992–1997; 1998–2002; 2003–September 15, 2008; and September 16, 2008–2012. Patients with CF with DM are shown with solid lines, and patients with CF without DM are shown with dashed lines. Females are shown in red and males in blue. Source Ref: [12].

The negative impact of a diagnosis of diabetes on survival in individuals with CF is well established. One large CF center which has been doing oral glucose tolerance screening since the early 1990′s prospectively assessed the mortality risk associated with the diagnosis of diabetes between 1992 and 2012 [10]. They found significantly greater mortality in individuals with CFRD compared to CF patients without diabetes; this difference improved over time but did not disappear. Between the years 1992 and 1997, the risk of death was 13.4 times higher in patients with CFRD compared to those without diabetes and was significantly worse in women [11]. Between the years 1998 and 2002, there was improvement in survival in the CFRD population, but risk of death was still 9 times higher in CF patients with diabetes compared to those without, with the greatest improvement seen in younger patients and men. In 2002–2008 mortality was 3.5 times higher in those with diabetes. The latest analysis from this center, covering the years 2008–2012, was not significantly different than the 2002–2008 analysis, and showed that the age adjusted mortality for patients with CFRD was 1.8 per 100 person-years, compared to 0.5 in patients with CF without diabetes (P = 0.0002), representing a 3.5-fold increase in per-person mortality in those with CFRD [12] (Fig. 2). After about 30 years of age, severe CFTR genotypes were associated with greater mortality compared to mild genotypes, although CFRD was associated with increased risk of death within each genotype category (20 vs 2%, p = 0.007 for mild; 12 vs 4%, p = 0.012 for severe), with greater mortality risk in females than males. The investigators speculated that the decline in diabetes-associated mortality over the years was due to increased OGTT screening resulting in earlier diagnosis and more aggressive treatment with insulin. They felt, however, that despite maximizing screening and insulin treatment, improvements in survival had “stalled” and that completely obliterating the survival discrepancy related to diabetes would require a different approach. They were hopeful that new CF modulator medications might have an impact.

### CFRD and lung function decline

While the main cause of death in patients with types 1 and 2 diabetes is cardiovascular disease, patients with CFRD, like all CF patients, primarily die from lung disease. CFRD accelerates the decline in lung function and increases the frequency of acute pulmonary infectious exacerbations [13], [14]. The proportion of patients with low or moderately low FEV1 (forced expiratory volume in 1 s) is significantly higher in all CF age groups in those with CFRD [15]. The degree of lung function loss is still not well defined but is estimated to be between 0.85 and 2.1% of the predicted FEV1 per year [16]. Of note, the trajectory of lung function decline becomes more rapid approximately 4 years before the diagnosis of CFRD, during the period when insulin insufficiency causes abnormal glucose tolerance but diagnostic criteria for diabetes have not yet been met [17]. Lung function is directly related to oral glucose tolerance status: when followed for 4 years after a baseline OGT test, patients with abnormal glucose tolerance had a greater loss of lung function than those with normal glucose tolerance, and those with early CFRD (normal fasting glucose) had the greatest loss [18]. In that study, loss of lung function was related to the degree of insulin insufficiency, measured as insulin area under the curve during an OGTT.

### By what mechanisms does CFRD negatively impact lung function and survival in CF?

There are two primary mechanisms by which diabetes is felt to accelerate the course of CF pulmonary disease: compromised nutrition due to an insulin insufficient catabolic state, and the negative impact of hyperglycemia on lung inflammation and infection.

Achieving and maintaining a normal body weight and lean body mass have long been known to be critical for maintaining good lung function in a patient with CF. Underweight and decreased lean body mass are associated with increased risk of death [19]. Several factors contribute to undernutrition in CF, including insulin insufficiency. Insulin is a known anabolic agent, and the insulin insufficiency seen in CFRD creates a catabolic state resulting in excessive protein and fat breakdown and significant weight loss [20], [21]. Low BMI is more prevalent in all age groups of CF patients with diabetes. Insulin replacement has been established as a treatment for reversing chronic weight loss in patients with early CFRD [22]. Patients with abnormal glucose tolerance (impaired glucose tolerance or indeterminate glycemia) have also been shown to have worse nutritional and pulmonary status [21]. There are studies underway to determine the impact of insulin therapy on protein turnover and clinical function in those populations. While insulin insufficiency and resultant protein catabolism are thought to be the primary explanation for higher mortality in patients with CFRD, the direct impact that hyperglycemia has on lung function likely also plays a role.

Hyperglycemia causes a pro-inflammatory state and an increase in oxidative stress [23]. Advanced glycation end products (AGEs) are formed through non-enzymatic Maillard reactions where proteins and lipids undergo irreversible glycation in the setting of hyperglycemia. The receptor for AGEs (RAGE) is expressed in the lungs [16]. In patients with type 1 and 2 diabetes, a significant decline in lung function has been reported as high as two to three times faster than in normal non-smoking subjects as reported in longitudinal studies [24]. The pulmonary changes described in individuals with type 1 and type 2 diabetes have seldom been reported to be of clinical consequence in those populations. However, it is reasonable to assume that the impact of subtle lung changes could be greater in someone who has severe underlying lung disease. In support of this idea, plasma levels of AGEs were about 10% higher in CF patients with diabetes compared to those without. A negative correlation was found in CF between plasma AGEs levels and pulmonary function, including both FEV1 and FVC percent predicted [25].

Hyperglycemia also has a negative effect on the pancreatic beta cell, as part of a self-perpetuating destructive cycle. In types 1 and 2 diabetes, beta cell loss leads to hyperglycemia, which, in turn, acutely increases circulating cytokine concentrations including interleukin-1β (IL-1β). IL-1β stimulates DNA damage in beta cells, in a process primarily medicated by nitric oxide. Further loss of beta leads to more hyperglycemia, which leads to more IL-1 β. Interleukin-1 β has been found in pancreatic autopsy samples from patients with CF, suggesting this pathway contributes to an accelerated rate of beta cell loss in CFRD [26].

In addition to promoting inflammation, hyperglycemia gives rise to a pro-infection state in the airways. Bacteria and various pathogens thrive in a hyperglycemic environment. Glucose, not typically present in airway secretions in normoglycemic individuals, does appear in the pulmonary secretions once plasma glucose levels are > 144 mg/dL (8.0 mmol/L) [27]. Insulin replacement therapy has been the mainstay of treatment for CFRD because it addresses both the protein catabolic state that results from insulin insufficiency, and the pro-inflammatory, pro-infectious state caused by hyperglycemia.

### CFRD and transplant prognosis

Lung transplantation is an accepted life-prolonging therapy for end-stage CF lung disease. 92% of solid organ transplants in CF are lung transplants [28]. Less commonly, liver transplantation is also performed in this population. Diabetes associated with transplantation can be categorized as either pre-existing before surgery, or diabetes that develops post-transplant.

Prior to transplant, 29–65% of CF patients have been described as having diabetes [29], [30]. Diabetes has been associated as a risk factor for death while waiting for a lung transplant, likely related to the underlying severity of illness in these patients [31]. Pre-existing diabetes has been variously described as increasing the risk of transplant morbidity and mortality or having no effect [32]. From these articles it appears that patients with pre-existing diabetes are at greatest risk of death in the 3 month peri/post-transplant period related to infection, bleeding and multiorgan failure; If they survive the first few months after transplant, mortality is likely no different than for CF patients without pre-existing diabetes. In the long term, transplant may improve diabetes control by reducing insulin resistance related to chronic lung infections and inflammation [33].

In the general, non-CF population, diabetes is a common complication following lung transplantation, with one study reporting that 32% of patients developed new-onset diabetes post-transplant [28]. Similarly, in CF, 20–63% of those not previously described as having CFRD developed diabetes [34]. This is related to several factors including baseline beta cell loss/dysfunction, post-operative stress, enteral nutrition, and beta-cell toxicity from immunosuppressive therapy.

There have been a handful of cases where islets or a pancreas were transplanted at the time of lung and or liver transplant [35], [36]. While this approach is intellectually appealing, the rareness of these combined transplant procedures is likely related to the complexity and the significant anesthesia time required just for the primary organ procedure, and to concerns about increased risk of rejection if multiple donors are required.

### CFRD and modulator therapy

One cannot review CFRD prognosis in this day and age without discussing CFTR modulators. These drugs target the basic CF defect to make cellular salt and water transport more physiologically normal by improving production, intracellular processing and/or function of the detective CFTR protein. They have revolutionized the care of patients with CF. While past treatments focused on slowing the inexorable decline in lung function, CFTR modulators actually improve FEV1 and reduce the number and severity of acute pulmonary infectious exacerbations, and patients report better symptom-related quality of life. CFTR modulators have had a particular impact on weight and BMI. CF has always been associated with underweight, with patients traditionally struggling to achieve and/or maintain a normal BMI and LBM. Weight gain is a prominent effect of CFTR modulators. We are in fact now seeing CF patients with higher BMIs and weight gain, and will possibly see a future trend towards obesity rates more similar to the general population. With this may come the propensity for the metabolic diseases that are becoming so prevalent in the rest of society.

There are thus far few specific data on the impact of CFTR modulators on either the incidence or severity of diabetes. For those patients with specific mutations that respond to the drug ivacaftor (about 4% of the CF population), small studies showed a positive impact on insulin secretion after 1 month and 4 months of Ivacaftor therapy [37]. The ivacaftor real world US and UK registry study showed a modestly reduced prevalence of CFRD in the ivacaftor group (30% vs 40% in the US, and 21% vs 29% in UK) [38]. In one study, 12 patients with normal to mildly impaired glucose tolerance treated with ivacaftor over 4 months had an improvement in arginine-induced c-peptide secretion consistent with a positive effect on beta cell and possibly alpha cell function.

Patients with more severe (and more typical) CF mutations were treated with the combination drug lumacaftor/ivacaftor. A US post-marketing study followed serial OGTTs for 1 year in about 40 patients treated with this medication [39]. Compared to baseline, OGTT fasting and 2-hour glucose levels, glucose area under the curve, insulin area under the curve and time to peak insulin level were not significantly different at 3, 6 and 12 months on lumacaftor/ivacaftor therapy. In contrast, a French study with a similar number of subjects, 58% of patients showed an improvement in oral glucose tolerance [39]. The two studies differed in that the French study had much narrower entrance criteria, excluding subjects with either existing diabetes or with normal glucose tolerance (Fig. 2). Since these studies, a more effective triple-modulator therapy drug has been introduced, and studies are underway to determine if this drug influences insulin secretion.

Most of the interest in CFTR modulator therapy in CFRD has focused on potential benefit, including whether it might prevent or delay onset of diabetes if started early enough in life, improve insulin secretion, and/or reduce CFRD-associated mortality. But modulators also have the potential to introduce new metabolic problems. They improve weight and BMI to the point that we are starting to see CF patients with obesity, which may place further stress on a reduced beta cell mass. It remains to be seen whether development of obesity in CF creates a clinical picture closer to type 2 diabetes.

## Conclusion

Diabetes has a negative impact on lung function and survival in CF which appears to have persisted over the last 3 decades; even as overall mortality has improved due to better treatment options in all patients with CF. The last few years have seen dramatic changes in CF medication therapy, and more data are needed to determine the impact of these newer drugs on CFRD prognosis.

### CRediT authorship contribution statement

**Zahrae Sandouk:** Conceptualization, Methodology, Resources, Writing – original draft. **Farah Khan:** Resources, Writing – original draft. **Swapnil Khare:** Resources, Writing – original draft. **Antoinette Moran:** Conceptualization, Methodology, Resources, Writing – review & editing, Supervision.
